# Factors Affecting the Quality of Life of Patients with Painful Spinal Bone Metastases

**DOI:** 10.3390/healthcare9111499

**Published:** 2021-11-03

**Authors:** Yoshiteru Akezaki, Eiji Nakata, Masato Kikuuchi, Shinsuke Sugihara, Yoshimi Katayama, Haruki Katayama, Masanori Hamada, Toshifumi Ozaki

**Affiliations:** 1 Division of Physical Therapy, Kochi Professional University of Rehabilitation, Kochi 781-1102, Japan; akezakiteru@yahoo.co.jp; 2 Department of Orthopaedic Surgery, Okayama University Hospital, Okayama 700-8558, Japan; misimaryou0313@gmail.com (H.K.); tozaki@md.okayama-u.ac.jp (T.O.); 3 Department of Rehabilitation Medicine, National Hospital Organization Shikoku Cancer Center, Ehime 791-0280, Japan; kikuuchi.masato.tu@mail.hosp.go.jp (M.K.); sugihara.shinsuke.rk@mail.hosp.go.jp (S.S.); 4 Department of Rehabilitation Medicine, Okayama University Hospital, Okayama 700-8558, Japan; yoshimikatayama@yahoo.co.jp (Y.K.); pjvy4uc1@okayama-u.ac.jp (M.H.)

**Keywords:** quality of life, spinal bone metastases, radiotherapy, activities of daily living, pain

## Abstract

This study examined changes in the quality of life (QOL), as well as the factors affecting QOL, among patients with painful spinal bone metastases without paralysis for 1 month after radiotherapy. Methods: This study included 79 participants (40 male and 39 female; median age, 65 (42–88) years) who had undergone radiotherapy for painful spinal bone metastases without paralysis. Patients’ age, sex, activities of daily living (Barthel index), pain, spinal instability (spinal instability neoplastic score [SINS]), and QOL (EORTC QLQ-C30) were investigated. Results: Having an unstable SINS score was a positive factor for global health status (*p* < 0.05). The improvement in activities of daily living and response to pain were positive factors for physical function (*p* < 0.05). A positive effect on emotional function was confirmed among female patients (*p* < 0.05). Conclusion: Engaging in rehabilitation along with radiotherapy leads to improvements in QOL for patients with spinal bone metastases.

## 1. Introduction

Bone metastases frequently occur in patients with advanced cancer [1,2]. The spine is the most common site of bone metastasis [1,2,3,4], with approximately 60–70% of advanced cancer patients developing spinal metastases during disease progression. Bone metastases progress gradually and can cause skeletal-related events (SREs), including malignant spinal cord compression, vertebral body fractures, and radiotherapy (RT), leading to painful vertebral metastases [3,5,6,7,8]. SREs are associated with reduced survival [9]. The survival rate of patients with bone metastases is increasing because of the development of effective treatment options, such as orthopedic interventions, drug treatments, and multidisciplinary approaches to cancer management [10].

Quality of life (QOL) is a multidimensional construct that includes physical, emotional, social, and functional domains of well-being [11,12]. The QOL of cancer survivors is worse than the QOL of non-cancerous patients [13,14]. Although reports on patients with spinal bone metastases have been limited, SREs are known to significantly reduce QOL [9,15]. Cancer-related pain has a negative impact on QOL as well as general activity, mood, walking ability, work, and overall enjoyment of life [16,17]. The main goal of treatment for patients with bone metastases is symptomatic pain relief and prolonged survival; however, maintaining or improving the patient’s QOL is also important [18,19,20].

RT has been shown to reduce pain and improve QOL [21]. Moreover, in advanced cancer patients with bone metastases undergoing palliative RT, the Karnofsky performance scale (KPS) and age were correlated with QOL [22]. Factors that affect the QOL of patients with spinal bone metastases may not only include pain but may also include factors such as activities of daily living (ADL). However, to the best of our knowledge, few studies have examined the factors affecting QOL in patients with spinal bone metastases. Understanding the factors that influence the QOL of patients with spinal bone metastases is important for providing treatment and care.

This study examined the changes in QOL and the factors affecting QOL in patients with painful spinal bone metastases for 1 month following RT.

## 2. Methods

### 2.1. Study Design

This was a retrospective, observational investigation of changes in the QOL of patients with spinal bone metastases.

### 2.2. Patients

The participants included 280 patients who underwent RT for painful spinal bone metastases without paralysis at our institution between July 2012 and December 2016. Among them, 79 patients (40 male and 39 female; median age, 65 (42–88) years) whose measurements were available before and 1 month after RT were investigated. The patients excluded from the research were those for whom clinical categories were missing.

Patients performed muscle strengthening exercises, balance exercises, and ADL exercises from an early stage in accordance with their condition.

### 2.3. Clinical Parameters

Patients’ age, sex, ADL, pain, spinal instability, and QOL were investigated.

### 2.4. Measurement of ADL

The Barthel index is a measure of the ability to perform ADL on a scale of 0–100 (0, very dependent; 100, independent) [23]. These items in the Barthel index assess a patient’s ability to perform feeding, bathing, grooming, dressing, bowel and bladder control, toileting, chair transfer, ambulation, and stair climbing.

Measurements were performed before and 1 month after RT. The patients whose scores improved or remained unchanged after 1 month were classified into the ADL improvement group, while the other patients were classified into the ADL non-improvement group.

### 2.5. Measurement of Pain

Based on the definition of IBMCWP, pain responses were classified as either a complete response (CR), a partial response (PR), pain progression (PP), or an indeterminate response (IR). Analgesic consumption was recorded, and all opioid analgesics were converted into the oral morphine equivalent dose (OMED) at each time point. CR was defined as a pain score of 0 at the treated site with no concomitant increase in analgesic intake (keeping stable or reducing analgesics in the daily OMED). PR was defined as pain reduction at the treated site of 2 or more points using a numerical rating scale of 0 to 10 without analgesic increase, or an analgesic reduction rate of 25% or more from baseline without an increase in pain. PP was defined as an increase in the pain score for the treated site of 2 or more points above the baseline value with a stable OMED, or an increase of 25% or more in the OMED compared with baseline with the pain score being stable or 1 point above baseline. IR was defined as any response that is not captured by the CR, PR, or PP definitions.

CR or PR was classified as the response group, and PP or IR was classified as the no-response group.

### 2.6. Measurement of Spinal Instability

Spinal instability was evaluated using the spinal instability neoplastic score (SINS) [24]. Based on the SINS criteria, patients were divided into three categories: those with stable (0–6 points), potentially unstable (7–12 points), and unstable (13–18 points) spines. In this study, spinal instability was classified into stable and unstable groups. The stable group included patients who were stable 1 month following RT. The unstable group included patients who were potentially unstable and unstable at 1 month following RT.

### 2.7. Measurement of QOL

The EORTC QLQ-C30 consists of one global domain (global health status), five functional domains (physical function, emotional function, social function, role function, and cognitive function), eight symptoms (fatigue, pain, nausea/vomiting, constipation, diarrhea, insomnia, dyspnea, and appetite loss), and financial impact. Our study used the five functional domains. For the global health and functioning domains, higher scores indicate higher QOL [25].

Measurements were performed before and 1, 2, and 3 months after RT. Patients whose global health and functioning domains improved by 10 points or more at 1 month after RT compared to before RT were classified into the improvement group, while the others were classified into the no improvement group.

### 2.8. Statistical Analysis

The QOL of the two sexes was examined using a chi-squared test. A comparison of QOL before RT and 1 month after RT, as well as before RT and 3 months after RT, was analyzed using the Wilcoxon signed-rank test.

To assess the factors affecting QOL at 1 month after RT, logistic regression analyses were conducted to determine which variables (i.e., age, sex, ADL, pain, spinal instability) were the best predictors of QOL at 1 month after RT. The SINS stable and unstable groups were compared, using the Wilcoxon signed-rank test, for changes in global health status before, 1 month after, and 3 months after RT, respectively.

Comparisons of QOL before and 1 month after RT and before and 3 months after RT, using the Wilcoxon signed-rank test, were used to adjust the *p*-values for multiple pairwise comparisons (*p* < 0.05/2 = 0.025, corrected for 2 pairwise comparisons). Other tests were two-sided, and statistical significance was set at *p* < 0.05. All analyses were conducted using SPSS software (version 22.0; IBM, Tokyo, Japan).

## 3. Results

### 3.1. Characteristics before RT and Pain, ADL, and Spinal Instability 1 Month after RT

The patients’ characteristics are shown in Table 1. All patients received RT. None of the patients underwent surgery.

One month after RT, pain responses were observed in 72 patients in the response group and 7 patients in the no-response group. ADL measurements were obtained for 65 patients in the improvement group and 14 patients in the no-improvement group. Spinal instability was observed among 37 patients in the stable group and 42 patients in the unstable group.

### 3.2. Comparison of QOL before and after RT

The comparison between QOL before and 1 month after RT is shown in Table 2 and Table 3. There was no significant difference in QOL between the sexes. Global health status and physical functioning showed significant improvement 1 and 3 months after RT compared to before RT (*p* < 0.05). Emotional functioning showed significant improvement 3 months after RT.

### 3.3. Factors Affecting Improvement in QOL 1 Month after RT

The results of the logistic regression analyses are shown in Table 4, Table 5 and Table 6. Being categorized in the SINS unstable group was a positive factor for global health status (*p* < 0.05). The improvement in ADL and response to pain were positive factors for physical function (*p* < 0.05). A positive effect on emotional function was confirmed in female patients (*p* < 0.05). For social, role, and cognitive functions, variables with significant differences were not extracted.

In the SINS unstable group, global health status and physical function improved 1 month after RT (Table 7).

## 4. Discussion

This study examined the changes in the QOL of patients with painful spinal bone metastases, as well as the factors affecting QOL, at 1 month following RT. Sex, ADL, pain, and spinal instability each had a strong influence on QOL following RT among patients with painful spinal bone metastases without paralysis.

Changes in QOL following RT were reported in the forms of immediate deteriorations in the physical and functional domains during the first week, as well as improvements in the psychosocial domain among patients with bone metastases [26]. Our study showed significant improvements in global health status and physical function after 1 month of RT. McCabe et al. found that the mean range of each item of the EORTC QLQC30 for patients with recurrent cancer (prostate, breast, colorectal, lung, pancreas, and others) was 49.5–59.9 for global health status, 63.7–77.7 for physical function, 63.6–71 for emotional function, 56.6–72 for social function, 54.6–72.5 for role function, and 72.8–78.5 for cognitive function [27]. Zeng et al. reported a baseline average value of 49.6 for global health status, 47.4 for physical function, 60.4 for emotional function, 52.7 for social function, 41.9 for role function, and 72.8 for cognitive function for patients with bone metastases [28]. Therefore, cancer patients with bone metastases may have reduced QOL. Similar results were noted in the current study; however, the patients showed significant improvement in global health status, physical function, and emotional function 3 months after RT. The emotional, social, role, and cognitive functioning aspects of QOL did not significantly improve. Thus, after RT, it is necessary to provide medical care while being cognizant of the components of QOL that are difficult to improve.

Being categorized in the unstable SINS group had a positive effect on global health status. However, the global health status and physical function of this group improved 1 month after RT.

The KPS had the greatest influence on the EORTC QLQ-C30 domain scores in advanced cancer patients referred for RT for the treatment of bone metastases [22]. All but three scales (nausea and vomiting, constipation, and diarrhea) of the QLQ-BM22 and QLQ-C30 had a moderate or better correlation with KPS in cancer patients with bone metastases [29]. SREs, pain, and Eastern Cooperative Oncology Group performance status were significantly related to lower EQ-5D scores in the multivariable analysis [30]. In this study, ADL affected physical function 1 month after RT. The effects of exercise therapy on bone metastases have been reported to improve physical function, physical activity levels, and lean mass in patients with prostate cancer [31]. Other studies have shown that inpatient rehabilitation improves ADL at discharge in patients with metastatic spinal cord compression [31,32]. Improvements in ADL may lead to an improvement in QOL in patients with spine metastases undergoing RT.

Pain is one of the most common symptoms of bone metastases, occurring in an estimated 68–70% of patients [33,34]. As a consequence of bone pain, patients often experience challenges in ADL and decreased QOL [33]. In this study, pain significantly affected physical functioning 1 month after RT; however, maximum pain relief was noted 4 weeks after RT, with 76% of patients having either partial or complete relief and 8.8% of patients having complete pain relief [9]. Other studies have reported that 17% of patients had complete pain relief and 49% of patients had partial pain relief 3 months after RT [35]. In this study, pain was found to have reduced 1 month after RT compared to rates before RT, suggesting that response to pain had a positive effect on physical function at 1 month after RT.

Female sex had a positive effect on global health status 1 month after RT. Male patients may have a lower QOL than females because they are usually responsible for the family’s finances and demand a high level of recovery.

### Study Limitations

There were limitations to the present study. This study was limited to factors affecting early QOL at 1 month after RT, and factors affecting long-term QOL were not examined. Moreover, patients in the present study were limited to those who had undergone RT; thus, our study cannot be compared with studies evaluating both patients who underwent surgery and those who did not. Further investigation is required to address these limitations.

## 5. Conclusions

The QOL of patients with painful spinal bone metastases showed a significant improvement in global health status and physical function at 1 and 3 months following RT, respectively. One month after RT, improvements in pain and ADL improved QOL. Engaging in rehabilitation along with RT leads to improvements in QOL for patients with spinal bone metastases.

## Figures and Tables

**Table 1:** Characteristics of the patients with bone metastases before radiotherapy.

Characteristic	Number/Median
Primary cancer site ^a^	
Breast	19
Lung	25
Prostate	10
Colorectal	8
Stomach	3
Others	14
Radiation site ^a^	
Cervical spine	7
Thoracic spine	38
Lumbar spine	34
Analgesic ^a^	
Yes	72
No	7
Radiotherapy dose ^a^	
8 Gy	1
20 Gy	9
27 Gy	1
30 Gy	58
36 Gy	1
37.5 Gy	1
40 Gy	8
Bone modifying agents ^a^	
Yes	75
No	4
Spinal instability ^a^	
Stable	15
Unstable	64
Numerical rating scale during movement (score) ^b^	5.0 (1–10)
Barthel index (score) ^b^	85.0 (5–100)

^a^ number; ^b^ median (minimum–maximum).

**Table 2:** Differences in quality of life between sexes.

Variable	Male	Female	*p*-Value
Global health status			0.37
Improvement group	16	20	
No-improvement group	24	19	
Physical function			0.173
Improvement group	20	13	
No-improvement group	20	26	
Emotional function			0.053
Improvement group	8	16	
No-improvement group	32	23	
Social function			0.474
Improvement group	11	14	
No-improvement group	29	25	
Role function			0.066
Improvement group	11	19	
No-improvement group	29	20	
Cognitive function			0.162
Improvement group	11	17	
No-improvement group	29	22	

Number.

**Table 3:** Comparisons between quality of life before and after radiotherapy.

Variable	Before	1 Month	2 Months	3 Months	*p*-Value ^a^	*p*-Value ^b^
Global health status (scores)	34.7 ± 24.8 (33.3)	43.5 ± 22.8 (50.0)	46.7 ± 24.4 (41.7)	49.4 ± 24.7 (50.0)	0.005	0.002
Physical function (scores)	44.8 ± 28.9 (46.6)	51.9 ± 26.7 (53.3)	56.3 ± 25.1 (60.0)	60.8 ± 27.8 (66.6)	0.022	0.008
Emotional function (scores)	66.8 ± 27.2 (75.0)	67.7 ± 23.0 (66.6)	72.2 ± 22.3 (75.0)	76.2 ± 19.2 (75.0)	0.794	0.018
Social function (scores)	65.4 ± 33.3 (66.6)	63.5 ± 29.1 (66.6)	65.0 ± 28.8 (66.6)	66.9 ± 27.6 (66.6)	0.480	0.544
Role function (scores)	41.1 ± 35.9 (50.0)	45.1 ± 32.5 (50.0)	47.6 ± 32.4 (50.0)	52.7 ± 30.8 (66.6)	0.259	0.407
Cognitive function (scores)	66.6 ± 27.2 (66.6)	65.6 ± 27.9 (66.6)	68.2 ± 21.7 (66.6)	70.3 ± 25.0 (66.6)	0.648	0.807

Mean ± standard deviation (median); ^a^ before vs. 1 month; ^b^ before vs. 3 months.

**Table 4:** Factors affecting global health status.

Variable	B	Standard Error	Odds Ratio (95% CI)	*p*-Value
Age	0.036	0.026	1.036 (0.984–1.092)	0.176
Sex	0.435	0.492	1.545 (0.589–4.055)	0.376
ADL	0.491	0.647	1.633 (0.460–5.803)	0.448
Pain response	1.256	0.944	3.513 (0.552–22.347)	0.183
Spinal instability	1.086	0.504	2.962 (1.103–7.956)	0.031

B, unstandardized coefficient; CI, confidence interval; ADL, activities of daily living.

**Table 5:** Factors affecting physical function.

Variable	B	Standard Error	Odds Ratio (95% CI)	*p*-Value
Age	0.006	0.027	1.006 (0.954–1.062)	0.814
Sex	−0.932	0.514	0.394 (0.144–1.078)	0.070
ADL	1.941	0.851	6.962 (1.313–36.919)	0.023
Pain response	2.511	1.193	12.313 (1.188–127.646)	0.035
Spinal instability	0.826	0.526	2.284 (0.815–6.403)	0.116

B, unstandardized coefficient; CI, confidence interval; ADL, activities of daily living.

**Table 6:** Factors affecting emotional function.

Variable	B	Standard Error	Odds Ratio (95% CI)	*p*-Value
Age	−0.008	0.028	0.992 (0.939–1.049)	0.788
Sex	1.099	0.545	3.001 (1.032–8.725)	0.044
ADL	0.321	0.696	1.379 (0.352–5.398)	0.645
Pain response	0.099	0.963	1.105 (0.167–7.286)	0.918
Spinal instability	0.935	0.549	2.546 (0.868–7.467)	0.089

B, unstandardized coefficient; CI, confidence interval; ADL, activities of daily living.

**Table 7:** Changes in global health status in the SINS stable and unstable groups.

Group	Before	1 Month	3 Months	*p*-Value ^a^	*p*-Value ^b^
Global health status					
Stable group	33.3 (0–83.3)	33.3 (0–83.3)	48.3 (33.3–83.3)	0.964	0.233
Unstable group	33.3 (0–83.3)	50.0 (0–91.7)	50.0 (0–100)	0.003	0.005
Physical function					
Stable group	66.6 (20.0–93.3)	60.0 (6.6–100)	60.0 (33.3–100)	0.432	0.351
Unstable group	40.0 (0–100)	53.3 (0–93.3)	66.6 (0–100)	0.005	0.002
Emotional function					
Stable group	75.0 (16.6–100)	66.6 (8.3–100)	83.3 (25.0–100)	0.423	0.677
Unstable group	70.8 (0–100)	66.6 (0–100)	75.0 (25.0–100)	0.486	0.015
Social function					
Stable group	66.6 (0–100)	66.6 (0–100)	66.6 (0–100)	0.529	0.752
Unstable group	66.6 (0–100)	66.6 (0–100)	66.6 (0–100)	0.614	0.397
Role function					
Stable group	50.0 (0–100)	50.0 (0–100)	58.3 (0–100)	0.512	0.600
Unstable group	41.7 (0–100)	41.7 (0–100)	66.6 (0–100)	0.349	0.586
Cognitive function					
Stable group	66.6 (0–100)	66.6 (0–100)	83.3 (16.6–100)	0.441	0.550
Unstable group	66.6 (0–100)	66.6 (0–100)	66.6 (0–100)	0.938	1.000

Median (minimum–maximum); ^a^ before vs. 1 month; ^b^ before vs. 3 months.

## Data Availability

The data presented in this study are available on request from the corresponding author. The data are not publicly available due to Participant’s personal information.
